# The use of eculizumab in *Capnocytophaga canimorsus* associated thrombotic microangiopathy: a case report

**DOI:** 10.1186/s12879-021-05789-2

**Published:** 2021-02-01

**Authors:** Magnus Holter Bjørkto, Andreas Barratt-Due, Ingvild Nordøy, Christina Dörje, Eivind Galteland, Andreas Lind, Abdulkarim Hilli, Pål Aukrust, Geir Mjøen

**Affiliations:** 1 Department of Transplant Medicine, Oslo University Hospital, Birch-Reichenwaldsgate 34, NO-0483 Oslo, Norway; 2 Division of Critical care and Emergencies, Oslo University Hospital, Oslo, Norway; 3 Section for Clinical Immunology and Infectious Diseases, Oslo University Hospital, Oslo, Norway; 4 Research Institute of Internal Medicine, Oslo University Hospital, Oslo, Norway; 5 Department of Haematology, Oslo University Hospital, Oslo, Norway; 6 Department of Microbiology, Oslo University Hospital, Oslo, Norway; 7 Department of Internal Medicine, Diakonhjemmet Hospital, Oslo, Norway

**Keywords:** Eculizumab, Thrombotic microangiopathy, Complement, Capnocytophaga canimorsus, Case report

## Abstract

**Background:**

The use of complement inhibition is well established for complement mediated thrombotic microangiopathy, but its role in secondary forms of thrombotic microangiopathy is debated. We here present a case of thrombotic microangiopathy triggered by *Capnocytophaga canimorsus*, illustrating the diagnostic difficulties in discriminating between different thrombotic microangiopathies, and the dilemmas regarding how to treat this disease entity.

**Case presentation:**

A previously healthy 56-year-old woman presented with fever and confusion. She was diagnosed with sepsis from *Capnocytophaga canimorsus* and thrombotic microangiopathy. Marked activation of both T-cells, endothelium and complement were documented. She was successfully treated with antimicrobial therapy, the complement inhibitor eculizumab and splenectomy. After several weeks, a heterozygote variant in complement factor B was localized, potentially implying the diagnosis of a complement mediated TMA over an isolated infection related TMA.

**Conclusions:**

We discuss the possible interactions between complement activation and other findings in severe infection and argue that complement inhibition proved beneficial to this patient’s rapid recovery.

## Background

Thrombotic microangiopathies (TMA) are a group of life-threatening conditions presenting with hemolysis and microcirculatory thrombosis [1, 2]. There are a plenitude of causes, including ADAMTS-13 dependent thrombotic thrombocytopenic purpura (TTP), shigatoxin-associated hemolytic uremic syndrome (HUS), complement mediated TMA (CM-TMA, also called atypical HUS/aHUS) and “secondary TMAs” that can be infection associated (IA-HUS), pregnancy related, drug induced or autoimmune, amongst others.

It is well known that several infections can cause TMA, i.e. IA-HUS [1, 2]. This is seen with microbes such as *Streptococcus pneumonia* and HIV infection. Infections may also trigger an underlying primary TMA (TTP, CM-TMA) [2]. *Capnocytophaga canimorsus* is a gram-negative rod commensal to the oral flora of cats and dogs and may cause human infection when transmitted from bites. Life-threatening infections due to this microbe with naturally low virulence occur primarily in patients with pre-existing disease or in patients working closely with these animals. Several reports on serious complications and outcome of *C.canimorsus* infection have previously been published [3–7], also in immunocompetent patients [3, 4], and including descriptions of TTP or aHUS treated with plasma exchange [4, 5]. One report [7] also includes treatment with eculizumab after successful sepsis treatment had not cured the patient’s encephalopathy.

To our knowledge, this is the first description of *C. canimorsus* associated TMA with verified complement activation, extensive inflammatory workup, and treatment with eculizumab.

## Case presentation

A previously healthy 56-year-old woman was admitted with fever and confusion. The three previous days she had experienced abdominal discomfort and some diarrhea, but no bloody stools. On admission she had a temperature of 36.9 °C, Glasgow Coma Scale score of 14, and elevated C-reactive protein (CRP). There was tenderness in the right upper abdomen, otherwise normal physical examination. Thrombocytopenia, leukopenia, and hemolytic anemia were noted. She had oliguric renal failure and a respiration frequency of 20 per minute needing oxygen supplement. Treatment for sepsis was initiated.

The next day (day 1) she was transferred to Oslo university hospital due to progressing renal failure in need of dialysis and a thrombotic microangiopathy warranting further diagnostics. She was confused, in respiratory distress requiring high flow oxygen therapy and developed petechial bleedings. Pleural end pericardial effusions were observed. Blood smear showed schistocytosis and anisocytosis, and few, but normal platelets. Bone marrow smear showed hypocellularity suggestive of toxic bone marrow failure. Computed tomography (CT) scan demonstrated a slightly enlarged spleen and swollen kidneys. No intracerebral pathology was demonstrated. A selection of laboratory values is presented in Tables 1, 2 and 3. 
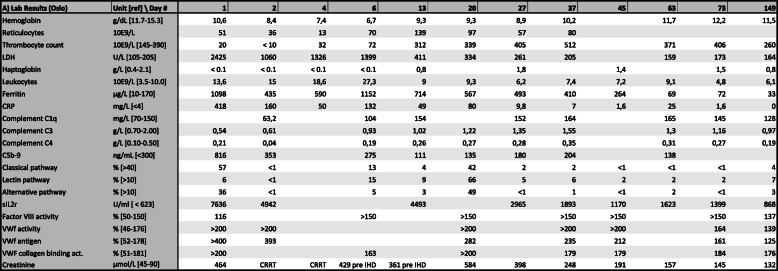

**Table 1:** Selected laboratory results from in-house laboratory

Lab Results (Oslo)	Unit [ref] \ Day #	1	2	4	6	13	20	27	37	45	63	73	149
Hemoglobin	g/dL [11.7-15.3]	10.6	8.4	7.4	6.7	9.3	9.3	8.9	10.2		11.7	12.2	11.5
Reticulocytes	10E9/L	51	36	13	70	139	97	57	80				
Thrombocyte count	10E9/L [145-390]	20	< 10	32	72	312	339	405	512		371	406	260
LDH	U/L [105-205]	2425	1060	1326	1399	411	334	261	205		159	173	164
Haptoglobin	g/L [0.4-2.1]	< 0.1	< 0.1	< 0.1	< 0.1	0.8		1.8		1.4		1.5	0.8
Leukocytes	10E9/L [3.5-10.0]	13.6	15	18.6	27.3	9	9.3	6.2	7.4	7.2	9.1	4.8	6.1
Ferritin	μg/L [10-170]	1098	435	590	1152	714	567	493	410	264	69	72	33
CRP	mg/L [<4]	418	160	50	132	49	80	9.8	7	1.6	25	1.6	0
Complement C1q	mg/L [70-150]		63.2		104	154		152	164		165	145	128
Complement C3	g/L [0.70-2.00]	0.54	0.61		0.93	1.02	1.22	1.35	1.55		1.3	1.16	0.97
Complement C4	g/L [0.10-0.50]	0.21	0.04		0.19	0.26	0.27	0.28	0.35		0.31	0.27	0.19
C5b-9	ng/mL [<300]	816	353		275	111	135	180	204		138		
Classical pathway	% [>40]	57	<1		13	4	42	2	2	<1	<1	<1	4
Lectin pathway	% [>10]	6	<1		15	9	66	5	6	2	2	2	7
Alternative pathway	% [>10]	36	<1		5	3	49	<1	1	<1	2	<1	3
sIL2r	U/ml [ < 623]	7636	4942			4493		2965	1893	1170	1623	1399	868
Factor VIII activity	% [50-150]	116			>150		>150		>150	>150		>150	137
VWf activity	% [46-176]	>200	>200				>200		>200	>200		164	139
VWf antigen	% [52-178]	>400	393				282		235	212		161	125
VWF collagen binding act.	% [51-181]	>200			163		>200		179	179		184	176
Creatinine	μmol/L [45-90]	464	CRRT	CRRT	429 pre IHD	361 pre IHD	584	398	248	191	157	145	132

*C5b-9* Soluble terminal complement complex, *CRP* C-reactive protein, *CRRT* continous renal replacement therapy, *IHD* intermittent hemodialysis, *LDH* Lactate dehydrogenase, *sIL* 2r Soluble interleukin 2 receptor, *VWf* von Willebrand factor


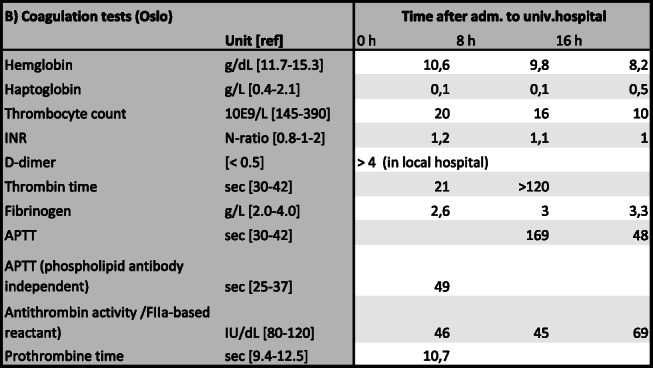

**Table 2:** Coagulation tests, detailed

Coagulation tests (Oslo)		Time after adm. to univ.hospital		
	Unit [ref]	0 h	8 h	16 h
Hemoglobin	g/dL [11.7-15.3]	10.6	9.8	8.2
Haptoglobin	g/L [0.4-2.1]	0.1	0.1	0.5
Thrombocyte count	10E9/L [145-390]	20	16	10
INR	N-ratio [0.8-1-2]	1.2	1.1	1
D-dimer	[< 0.5]	> 4 (in local hospital)		
Thrombin time	sec [30-42]	21	>120	
Fibrinogen	g/L [2.0-4.0]	2.6	3	3.3
APTT	sec [30-42]		169	48
APTT (phospholipid antibody independent)	sec [25-37]	49		
Antithrombin activity /FIIa-based reactant)	IU/dL [80-120]	46	45	69
Prothrombin time	sec [9.4-12.5]	10.7		

*APTT* Activated prothrombin time, *INR* International normalized ratio


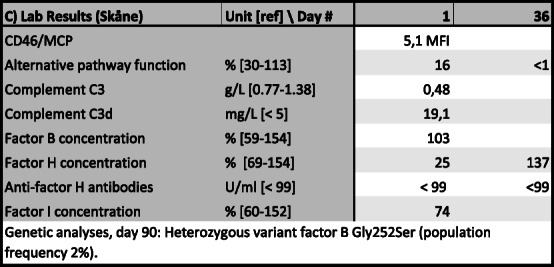

**Table 3:** Results from extended complement analyses, Skåne laboratory, Lund University, Sweden

Lab Results (Skåne)	Unit [ref] \ Day #	1	36
CD46/MCP		5,1 MFI	
Alternative pathway function	% [30-113]	16	<1
Complement C3	g/L [0.77-1.38]	0.48	
Complement C3d	mg/L [< 5]	19.1	
Factor B concentration	% [59-154]	103	
Factor H concentration	% [69-154]	25	137
Anti-factor H antibodies	U/ml [< 99]	< 99	<99
Factor I concentration	% [60-152]	74	

Genetic analyses, day 90: Heterozygous variant factor B Gly252Ser (population frequency 2%)

The clinical picture suggested systemic infection with secondary TMA, accompanied by cerebral confusion, respiratory impairment, and acute renal failure. Thrombotic Thrombocytopenic Purpura (TTP), Disseminated Intravascular Coagulation (DIC) and complement mediated TMA were possible differential diagnoses.

Initial treatment included IV meropenem 1 g twice daily, IV linezolide 600 mg twice daily and continuous veno-venous hemodialysis. Suspecting TTP, corticosteroids and plasma exchange (PE) were initiated, but were discontinued 24 h after admission, when we received data on ADAMTS13 [a Disintegrin And Metalloprotease with a ThromboSpondin type 1 motif, member 13] activity, which was within normal limits (> 70%), excluding TTP. Differential diagnosis of DIC as part of severe bacterial infection was less likely, since she was circulatory stable and had elevated rather that decreased levels of fibrinogen.

**Fig. 1:**
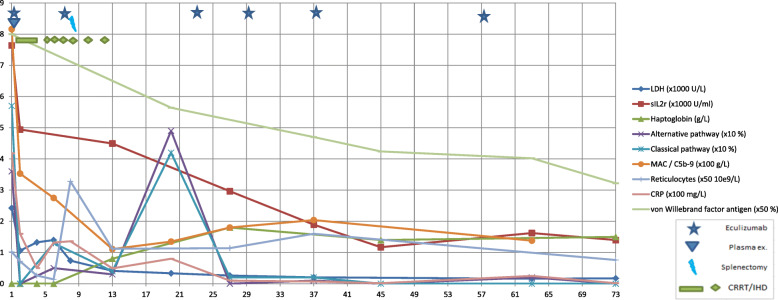
Selected laboratory parameters and clinical interventions. C5b-9 soluble terminal complement complex; CRP C-reactive protein; CRRT continuous renal replacement therapy; IHD intermittent hemodialysis; MAC membrane attack complex; LDH Lactate dehydrogenase; sIL2r soluble interleukin 2 receptor

Within the first day, low levels of complement factors C1q, C3 and C4 were detected, accompanied by an elevated level of soluble terminal complement complex sC5b-9 and diminished Factor H (Fig. 1, Tables 1, 2 and 3). There were also markedly elevated levels of von Willebrand factor (vWf) and soluble interleukin 2 receptor (sIL2R) suggesting activation of endothelial cells and T cells, respectively. Since we could not exclude a diagnosis of complement mediated TMA, treatment with the complement C5 inhibitor eculizumab was initiated with a starting dose of 900 mg.

During the first week, extensive diagnostics were performed. *E. coli* derived shigatoxin and streptococcal infection were excluded. Blood-cultures, viral DNA PCR (Cytomegalovirus, Epstein Barr virus, Influenza virus A and B) and antigen/serology tests (HIV, Hepatitis A, B and C) were all negative. Legionella and pneumococcal antigen in urine were negative. Autoimmune disease was unlikely based on negative autoantibodies (anti-nuclear antibodies, anti-granulocyte cytoplasmatic antibodies, lupus anticoagulant, antiphospholipid-antibodies and anti-glomerular basement membrane- antibodies). There was no evidence of malignancy based on findings in bone-marrow biopsy, CT and Positron emission tomography (PET) CT scans.

**Fig. 2:**
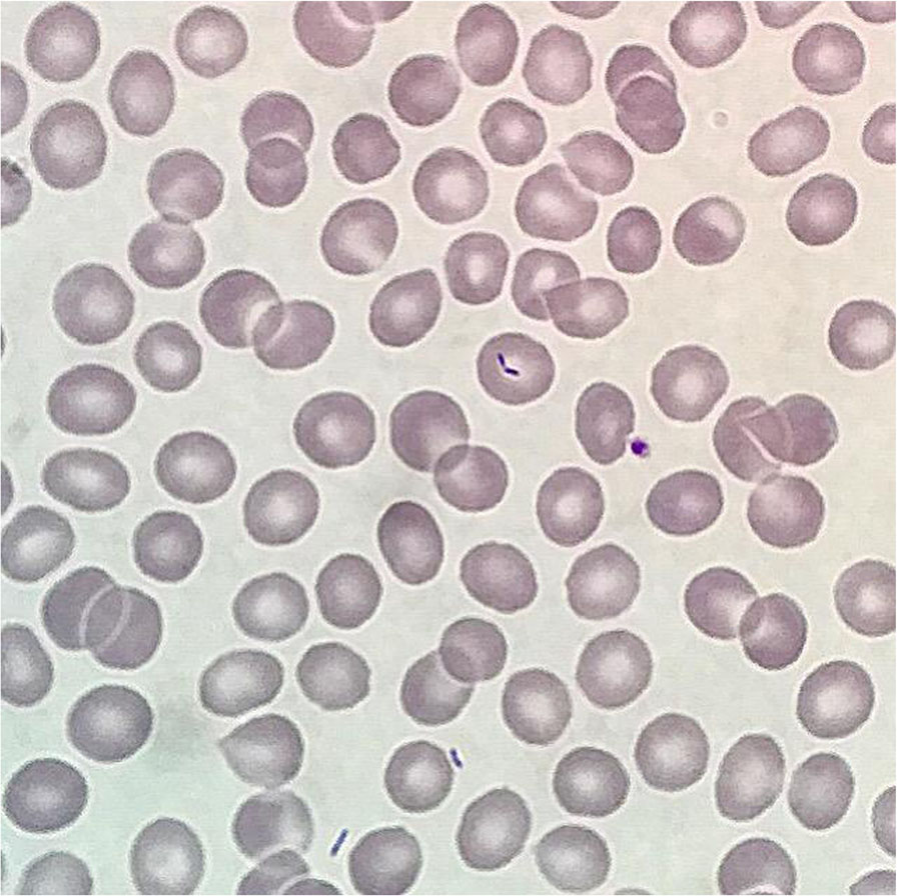
Rod-shaped bacteriae on hematological blood smear. This somewhat unusual finding was the first objective sign of a gram-negative sepsis and warranted continued treatment for such. Courtesy of dr. Hilli, Diakonhjemmet Hospital, Oslo, Norway

On Day 2, Rod-shaped bacteriae were suspected upon reexamination of initial blood smear (Fig. 2) at the primary hospital. On day 4 there was growth in blood cultures taken on first admission. This led to the suspicion of a slow-growing bacteria such as *C. canimorsus*. The patient had a dog, but no recent history of bites. Based on this finding, meropenem was continued, linezolid stopped, and clindamycin 900 mg three times daily added to the regimen. On Day 5, *C. canimorsus* was confirmed by mass spectrometry.

The patient recovered cognitively on Day 2 and physically on Day 4, when she was transferred to the medical ward. However, on day 5, a rise in CRP and leukocytes occurred. Ultrasonography demonstrated impaired splenic blood flow. On day 8 an open splenectomy was performed. Histological examination showed a necrotic spleen with signs of TMA and *C. canimorsus* was demonstrated by polymerase chain reaction (PCR). Based upon the possibility of complement-driven TMA, eculizumab injections were repeated after 7, 23, 30 and 38 days (doses of 900 mg). The planned dose on day 14 was withheld due to completely suppressed complement activation pathways.

Following splenectomy, the patient rapidly recovered. Dialysis was tapered and last session given on day 12. Meropenem was discontinued on Day 13, clindamycin on day 17. The patient was discharged after 30 days. She had not experienced any significant side effects from the treatment. During outpatient follow-up over the next months, she further regained her premorbid health status, except for a slightly reduced renal function. Eculizumab was continued in doses of 1400 mg every 3 weeks for 3 months. Genetic analyses found a heterozygous variant factor B Gly252Ser, associated according to Osborne with complement mediated TMA in a few cases and with a reported prevalence of 2% in the population according to the ExAC database. Eculizumab was then discontinued, after a total of 7 doses.

## Discussion and conclusions

*C. canimorsus* grows slowly. Consequently, it may take time to establish the proper diagnosis when TMA occurs. Other causes for TMA than IA-HUS must be excluded, since occult “primary” causes of TMA require unique therapies that could influence outcome. TTP and CM-TMA are two of the important differentials that can be specifically targeted. In our hospital, both TTP diagnostics and broad complement diagnostics take time since they need to be analyzed elsewhere. The dilemma is how to treat while awaiting the results.

The complement system is important in tissue homeostasis and immune surveillance, but overwhelming complement activation may contribute to destructive inflammation. Being part of the innate immune system, complement constitutes a first-line defense mechanism against various microbes including *Capnocytophaga* spp. [8]. *C.canimorsus* is a capsule-forming bacterium [9]. Capsules are a common way for microbes to evade the host immune defense because it makes the pathogen less vulnerable to phagocytosis. This might increase the risk of exacerbating bacteremia when complement is blocked by eculizumab. Still, excessive complement activation may mediate harmful effects on the host also during severe acute infections like septicemia, and these risks must be weighed. Targeted antibiotic therapy must at least be given parallel to eculizumab.

Complement activation in severe infections is a part of the physiologic response through several mechanisms. Massive complement activation is not necessarily deleterious, but could still be harmful. One could think that in the setting of infection with manifest TMA, complement inhibition could be beneficial to the patient even in the absence of pathologic complement defects. However, reviewing the literature on both secondary TMAs and (shigatoxin-related) HUS, the role of C5 inhibition is uncertain [10–13]. Then again, the diagnostics are complex, and different mediators of TMA may be active at the same time, as this case illustrates.

In the present case, we hypothesize that *C. canimorsus* infection triggered the harmful process leading to TMA, whereas complement activation exaggerated the systemic process inflicting damage to the kidney and spleen. The patient had a heterozygous factor B variant with a population frequency of approximately 2%, mentioned by Osborne with a possible association to CM-TMA [14], though at the same time classified as “likely benign”. Although factor B level was within normal limits, she certainly had an excessive complement activation, with low levels of factor H and C3 and markedly elevated levels of C3d. This rapidly normalized after eculizumab treatment. It is possible that the variant factor B puts the patient at risk of excessive complement activation in the presence of a trigger such as sepsis, but we do not believe her to be at risk of spontaneous TMA episodes.

Severe infections are often associated with endothelial cell activation reflected in elevated plasma levels of vWF. Recent studies suggest that vWF is a potent activator of the alternative pathway of complement activation [15]. Complement activation in our case was accompanied by markedly and persistently elevated levels of vWF, indicating that this could be a contributing mechanism of TMA development in our patient.

Factor H is an important regulator of the alternative complement activation pathway, exerting a negative feedback on C3 activation. Data on the regulation of factor H during severe inflammatory conditions including severe infections are scarce, but knowledge of the larger and multifunctional family of factor H related proteins is emerging [16]. Some of these may mediate effects opposing factor H itself. Furthermore, several microbes are known to actively bind factor H to evade the host complement system [17]. Finally, factor H levels could be actively suppressed by the host through unknown mechanisms related to severe inflammation. Regardless of the mechanism, decreased Factor H seems associated with the enhanced systemic complement activation in this patient.

T cell activation interacting with complement activation has been implicated in the pathogenesis of complement mediated kidney disease [18, 19]. This interaction has also been suggested in IA-HUS. Our patient was characterized by enhanced and persistently elevated levels of sIL2R, even when other parameters were nearly normalized. This may render a role of T cell activation in the pathogenesis of IA-HUS.

Limitations of this case report include the lack of previous medical data, whereby the patient might have undergone previous, subclinical TMA episodes. Also, at the time of eculizumab administration she was already treated with antibiotics and the fever was abating. She was however still cognitively affected, but the short time between different interventions makes it difficult to evaluate the eculizumab specific effect. Sokol et al. [7] describes a similar case, but had a longer interval between sepsis treatment and eculizumab administration, thus demonstrating better the latter’s effect on cognitive improvement. Strengths of our case include long follow-up, genetic analyses regarding complement and a broad inflammatory workup.

In conclusion, this case of *C. canimorsus* triggered TMA was characterized by enhanced activation of the complement system, endothelial cells and T cells. A complement factor B variant was identified, possibly facilitating excessive complement activation. We discuss other mechanisms through which severe infections might cause complement activation. We believe the patient benefited from complement inhibition by eculizumab, but are not able to conclude decisively. With normal complement levels after resolution of the infection, we do not believe the patient is at risk of spontaneous episodes of TMA, and chose discontinuation of eculizumab in the long term follow-up.

## Data Availability

All data analyzed in this study are included in this published article. Additional data generated during the treatment of the patient is not publicly available due to privacy matters. Additional clinical data can be made available upon reasonable request.

## Abbreviations

ADAMTS13: A Disintegrin And Metalloprotease with a ThromboSpondin type 1 motif, member 13
aHUS: Atypical hemolytic-uremic syndrome
CM-TMA: Complement mediated thrombotic microangiopathy
CT: Computed tomography
DIC: Disseminated intravascular coagulation
HUS: Hemolytic-uremic syndrome
IA-HUS: Infection associated hemolytic-uremic syndrome
PE: Plasma exchange
sIL2R: Soluble interleukin-2-receptor
TMA: Thrombotic microangiopathy
TTP: Thrombotic thrombocytopenic purpura
vWf: Von Willebrand factor
